# Direct healthcare cost of pediatric systemic lupus erythematosus in the Philippines

**DOI:** 10.3389/fped.2023.1166974

**Published:** 2023-05-30

**Authors:** Maria Kathryn Ramirez Yee, Ma Theresa Moreno Collante, Christine Bea Bernal

**Affiliations:** Section of Pediatric Rheumatology, Department of Pediatrics, University of Santo Tomas Hospital, Manila, Philippines

**Keywords:** pediatric, systemic lupus erythematosus, cost of illness, direct cost, Philippines, healthcare expenditure, lupus, healthcare cost

## Abstract

**Background:**

Pediatric rheumatic diseases are chronic illnesses that pose a huge economic burden to children and their families; one of the most common is pediatric systemic lupus erythematosus (pSLE). The direct cost of pSLE has been studied in other countries. In the Philippines, this was only studied in the adult population. This study aimed to determine the direct cost of pSLE in the Philippines and its cost predictors.

**Methods:**

A total of 100 pSLE patients were seen from November 2017 to January 2018 at the University of Santo Tomas. Informed consent and assent forms were obtained. A total of 79 patients met the inclusion criteria and parents were asked to answer a questionnaire. Data were tabulated and were subjected to statistical analysis. Cost predictors were estimated using a stepwise log linear regression.

**Results:**

A total of 79 pediatric SLE patients, with a mean age of 14.68 ± 3.24 years, 89.9% of which were females, with a mean disease duration of 36.08 ± 23.54 months, were included in this study. A total of 65.82% had lupus nephritis and 49.37% were in flare. The mean annual direct cost for pediatric SLE patient was 162,764.81 PHP (USD 3,047.23). Majority of the expense was for medications. Regression analysis showed that the predictors of increased cost in doctor's fee in clinic visits (*p*-value 0.000) and IV infusion (*p*-value 0.01) were the higher combined income of the parents.

**Conclusion:**

This is a preliminary study on the mean annual direct cost of pediatric SLE patients in a single center in the Philippines. Pediatric SLE patients with nephritis and other target organ damage were seen to increase the cost up to 2–3.5×. Patients in flare also had a higher cost of up to 1.6×. The overall cost driver of this study was the parent's or caregivers combined income. Further analysis showed that cost drivers in the subcategories include the age, sex and parent's/caregiver's educational attainment.

## Introduction

Pediatric rheumatic diseases are chronic illnesses that pose a burden to children and their families, the most common of which is pediatric systemic lupus erythematosus (SLE), which accounts for 15%–20% of the total population (1). It is a chronic, relapsing–remitting, multisystemic autoimmune inflammatory disorder. If not recognized early and addressed immediately, SLE may lead to irreversible complications like end organ damage such as end-stage renal disease, infection, and death (1). Follow-up and regular intake of medications is important in achieving and maintaining remission. Currently, new therapeutic options like biologic disease-modifying anti rheumatic drugs (DMARDs) have been made available in the country, which provide alternative and better control of their disease; however, this entails increased cost making treatment inaccessible and compliance difficult. This causes a significant impact on the quality of life of patients as well as their everyday cost of living.

In the Philippines, healthcare is financed by four sources, namely, the national and local government, both government and private insurance, out of pocket, and from donors. In 2007, the Department of Health noted that majority of the expense comes from out of pocket at 57% (2). In 2018, the Philippines government has passed the Comprehensive Lupus Prevention Act of 2018, which aimed to increase recognition, understanding, and awareness of SLE. Provision of fees for early detection and screening has been included in the bill (3). While detection is important, regular follow-up and adequate treatment are essential. Hence, out of pocket expense for maintenance medication remains to be burdensome for this group of patients.

The aim of this study is to determine the burden of pediatric SLE by estimating the mean annual direct cost and to identify the potential cost drivers.

## Materials and methods

### Participants

All pediatric SLE patients seen at the University of Santo Tomas Hospital clinical and private division from November 2017 to January 2018 who fulfilled the ACR classification criteria for pediatric SLE seen in the University of Santo Tomas OPD, private clinic, and Joint and Bone Center and has been diagnosed for more than a year were included in this study. Patients with other comorbid disease aside from this pediatric rheumatologic condition like cardiac and pulmonary disease not related to this illness, those included in clinical trials, newly diagnosed Lupus patients (less than 1 year), and intellectually disabled or patients with developmental delay were excluded from the study. Participants who did not complete the questionnaire were withdrawn from the study.

### Description of study procedure

Approval for the study was first obtained from the Institutional Review Board (IRB) of the University of Santo Tomas Hospital. After obtaining IRB approval, permission to conduct the study in the Outpatient and Private Division was sent to the head of the Outpatient Department and the Chair of the Department of Pediatrics. Letter of request to conduct the study in the private clinic was also sent.

The survey form consisted of two parts. The first part contained questions regarding the demographic data of the patient, particularly age and sex; illness particularly at the age at diagnosis, year of diagnosis, current disease status, type of organ involvement, current medications or infusions, frequency of ER visits and clinic consults, and monthly healthcare expenditure, particularly the amount spent on consults, hospitalizations, ER visits, IV infusions, medications, laboratory tests, and other ancillaries. Questions pertaining to the source of medical funds and compliance to medical treatment were also asked. The second part of the questionnaire was about the parent or caregiver's demographic data and socioeconomic data, particularly monthly income and percentage of the monthly household budget allocated for medical expense of their child, as well as their family profile.

Patients seen in the outpatient department, Joint and Bone Center, and private division with pediatric SLE from November 2017 to January 2018 were included in the study. Informed consent from the subject's parents and assent form from the subjects were obtained.

Instructions in answering the questionnaire was explained by the researcher, after which the subject's parents or caregivers were asked to answer and complete the questionnaire. The researcher was present all throughout and was available to assist the participants in answering the questions.

All the data obtained were tabulated. Data collected were then subjected to statistical analysis.

### Cost calculation

Direct healthcare costs were calculated for each patient. Healthcare costs were determined through the use of each patient's reported inpatient and outpatient expenditures incurred from the past year. Inpatient expenditures included the number of emergency room visits per year and the monthly cost per emergency room consult, as well as the number of admissions and cost per hospitalization. For outpatient expenditures, the number of physician consults and cost per consult and medication and laboratory expenses were also included. Medical expense per month for maintenance medications, oral immunosuppressive medications like mycophenolate mofetil, and IV infusions like cyclophosphamide, rituximab, and pulse steroids were also asked.

### Statistical analysis

Descriptive analysis and cross tabulation of demographic and socioeconomic data were done. Frequency was used for nominal variables. Mean and SD were used for interval variables. Cost data were presented as mean. Univariate analyses and stepwise log linear regression analysis were used to determine cost predictors. The constant variables used were the demographic and disease characteristics of the patient and the demographic profile of the parent or caregiver. A stepwise method was used to determine the factors related to the increase in cost. Microsoft Excel and SPSS 22 were used for the statistical analysis.

## Results

### Demographics and clinical profile

**Figure 1:**
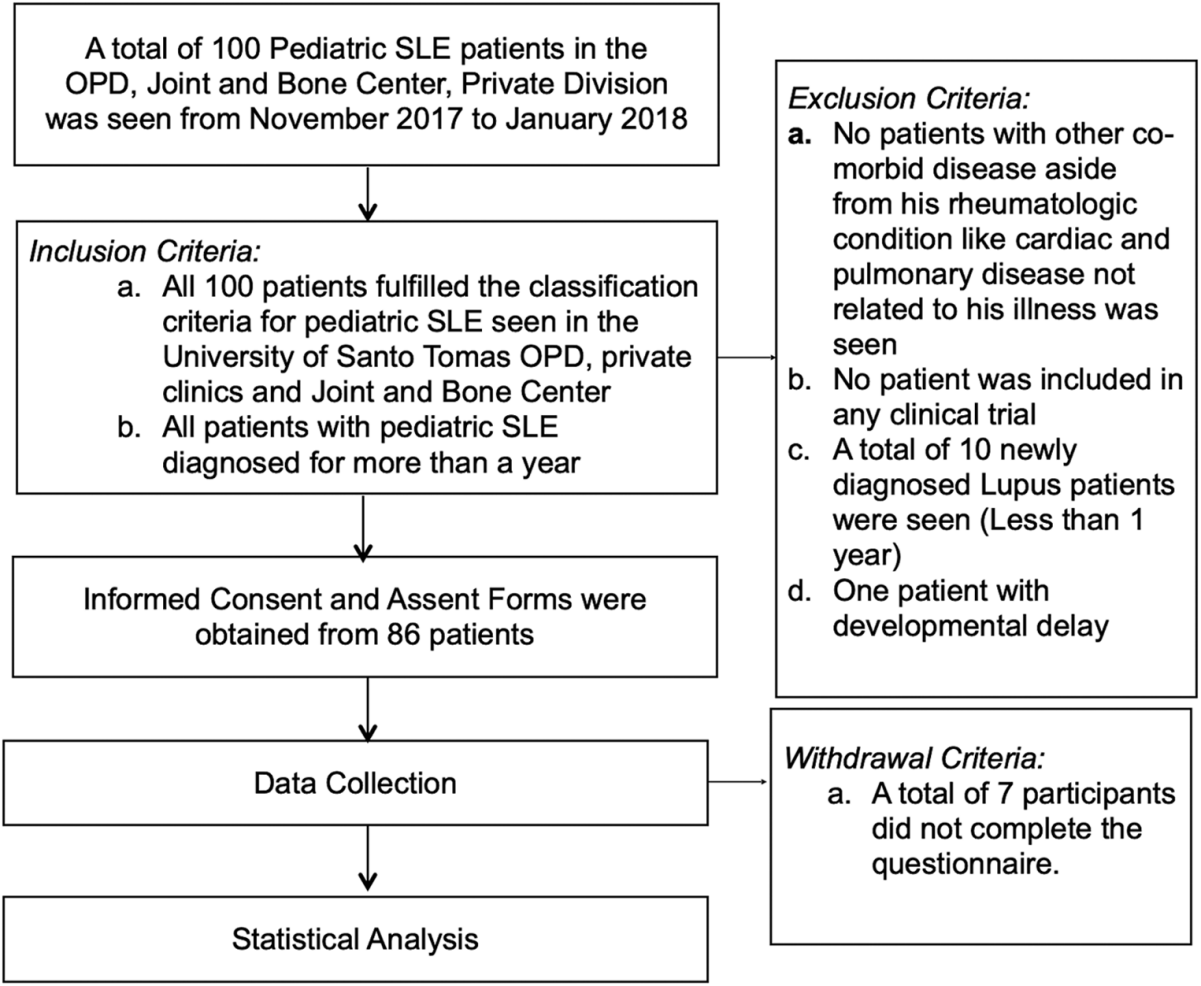
Overview of the study procedure.

The overview of the study procedure is summarized in Figure 1. From November 2017 until January 2018, a total of 100 pediatric SLE patients were seen in the Pediatric Rheumatology Department of the University of Santo Tomas Hospital, of which 79 were included in this study. Twenty-one patients were not included because 10 were newly diagnosed patients, 3 did not consent, 7 did not complete the questionnaire, and 1 had developmental delay.

The demographic and clinical profiles of pediatric SLE patients are summarized in Table 1. Pediatric patients with SLE, aged 7–19 years with a mean age of 14.68 ± 3.24 (median 15.00) were included, of which 71 were female and eight were male. Almost half of the patients at 49.37% were in flare. In this study, 83.54% had some form of organ involvement, of which 10.6% had more than one organ involved. The most common organ involved is the kidney where 65.82% had lupus nephritis. The mean disease duration is 36.08 ± 23.54 months (median 26.00).

**Table 1:** Demographic and clinical profile of pediatric SLE patients.

Demographic categories	Frequency (*N* = 79)	Percentage
Gender		
Female	71	89.87
Male	8	10.13
Age		
10 and below	9	11.39
11–12	11	13.92
13–14	16	20.25
15–16	16	20.25
17–18	19	24.05
19	8	10.13
Status of SLE		
Not in flare	40	50.63
In flare	39	49.37
Target organ involved		
Nephritis	52	65.82
Hematologic	4	5.06
CNS	2	2.53
GI	1	1.27
More than one organ	7	8.86
None	13	16.46
Disease duration (in months)		
12	8	10.13
13–24	28	35.44
25–36	14	17.72
37–48	8	10.13
49–60	9	11.39
61 and above	12	15.19

SLE, systemic lupus erythematosus; CNS, central nervous system; GI, gastrointestinal.

Pediatric SLE patients are mostly maintained on medications like glucocorticoids and hydroxychloroquine. Those with target organ involvement or difficult to manage SLE are given additional immunosuppressive medications. In our study population, 21.51% are on oral immunosuppressive medication (*n* = 17), while 53.16% of the patients are on intravenous infusions like cyclophosphamide (*n* = 42). Only 5% of the 79 patients were given newer biologic medications like rituximab. Compliance to medications according to the respondents was at 60.76%. In the past year, only 39.24% of the patients have been hospitalized and have visited the emergency room. The mean number of rheumatologic consults during the past year was 8.77, while the mean number of non-rheumatologic consults was at 1.8.

SLE being more common in females, results of this study showed a higher number of pediatric female patients with SLE. Among the 71 female patients included in this study, 47% were in flare. Target organ damage in the female pediatric SLE patients was present in 77.22%, the most common being nephritis. In the female population, 64.78% were on some form of immunosuppressive medication.

Among the eight male patients included in this study, all had nephritis and 62.5% of them were in flare and are currently being given a combination of immunosuppressive medication. Four out of the eight male patients have been hospitalized at least once in the past year.

Table 2 shows the demographic and socioeconomic profile of the parents/guardians of the pediatric SLE patients included in the study. The mean age of the parents/guardians is 44.27 ± 7.23 years (median 45.00), and majority were married (78.49%). The highest educational attainment of the parents/guardians is college at 51.9%, while 37.97% are high-school graduates; the remaining were elementary graduates. In most of the families, majority of the parents are employed, and among them, 48.10% of the parents are both working to sustain the needs of the family. Overall, a large part of the parents/guardians included in this study are educated and are working. Sources of medical funds were ranked and results showed that majority of their funds were out of pocket.

**Table 2:** Demographic profile of the parents/guardians of pediatric SLE patients.

Demographic categories	Frequency (*N* = 79)	Percentage
Age		
35 and below	7	8.86
36–40	17	21.52
41–45	20	25.32
46–50	21	26.58
51 and above	14	17.72
Civil status		
Single	7	8.86
Married	62	78.49
Widow	4	5.06
Separated	6	7.59
Educational attainment		
Elementary	8	10.13
High school graduate	30	37.97
College graduate	41	51.90
Employment		
Employed	75	94.9
Unemployed	4	5.1
Monthly household income		
≤8,000	13	16.46
8,000–15,000	26	32.91
15,000–30,000	18	22.78
30,000–50,000	12	15.19
50,000–100,000	8	10.13
≥100,000	2	2.53

SLE, systemic lupus erythematosus.

The percentage of monthly household income allotted for pediatric patients with SLE is illustrated in Table 3. In majority of the families, less than 25% of the income is allotted for their child's medical expense (41.77%), while less than 50% of the monthly budget is allotted by 31.65% of the families. Despite this, only 15.19% miss their medication most of the time due to lack of funds, while 24.05% only miss their medications once a week at most.

**Table 3:** Percentage of monthly household budget for child's medical expense.

Percentage of monthly household budget	Frequency (*N* = 79)	Percentage
<10%	10	12.66
<25%	33	41.77
<50%	25	31.65
≥50%	11	13.92

### Direct cost of pediatric SLE patients

The mean annual direct cost per patient with pediatric systemic lupus erythematosus in a single center in the Philippines is 162,764.81 PHP (USD 3,047.23).

**Figure 2:**
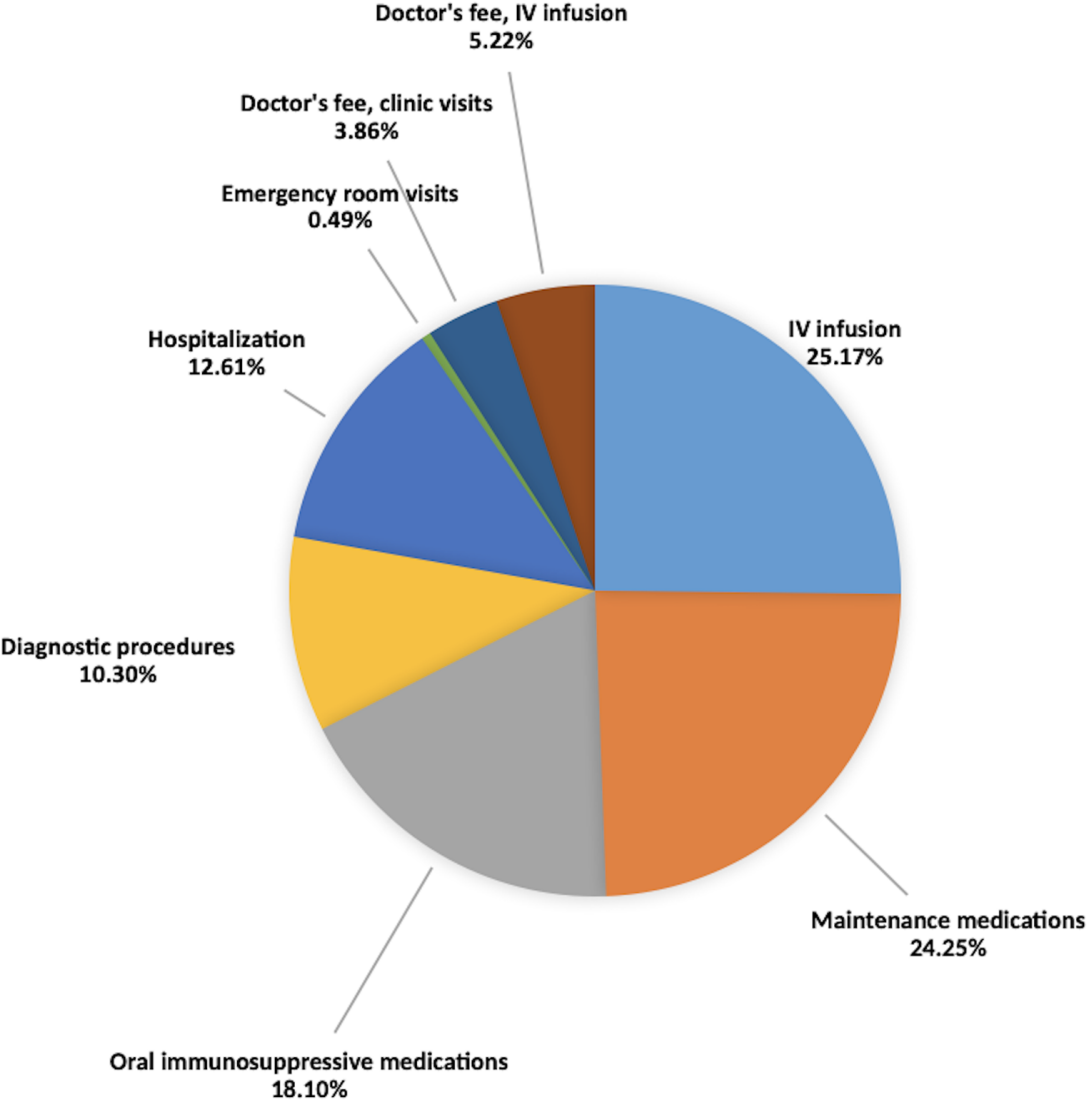
Components of healthcare cost.

Table 4 summarizes the mean annual direct cost per component in PHP. Figure 2 illustrates the percentage of each component per healthcare expenditure. Majority of the expense comes from medications at 67.52%, from which IV infusions are accounted at 25.17%, followed by maintenance medications at 24.25% and oral immunosuppressive medications at 18.10%. Diagnostic procedures accounted for 10.3%. Percentage allotted for hospitalization and emergency room visits are 12.61% and 0.49%, respectively. For the physician's fees, 3.86% are allotted for clinic visits while 5.22% are allotted for the physician's fee during IV infusions.

**Table 4:** Mean annual direct medical costs in pediatric SLE.

Mean annual direct medical costs	Overall Mean + SD (in PHP)
Maintenance medications	39,478.44 ± 27,294.69
Oral immunosuppressive medication expense	29,454.96 ± 67,333.45
IV infusion/pulse therapy	40,963.10 ± 27,674.50
Hospitalizations	20,518.99 ± 45,440.54
Emergency room visits	800.00 ± 1,604.99
Laboratory tests/ancillary procedures	16,773.12 ± 9,295.31
Doctor's fee, per IV infusion	8,496.20 ± 2,855.09
Doctor's fee, per clinic visit	6,280.00 ± 3,903.04

SLE, systemic lupus erythematosus.

**Figure 3:**
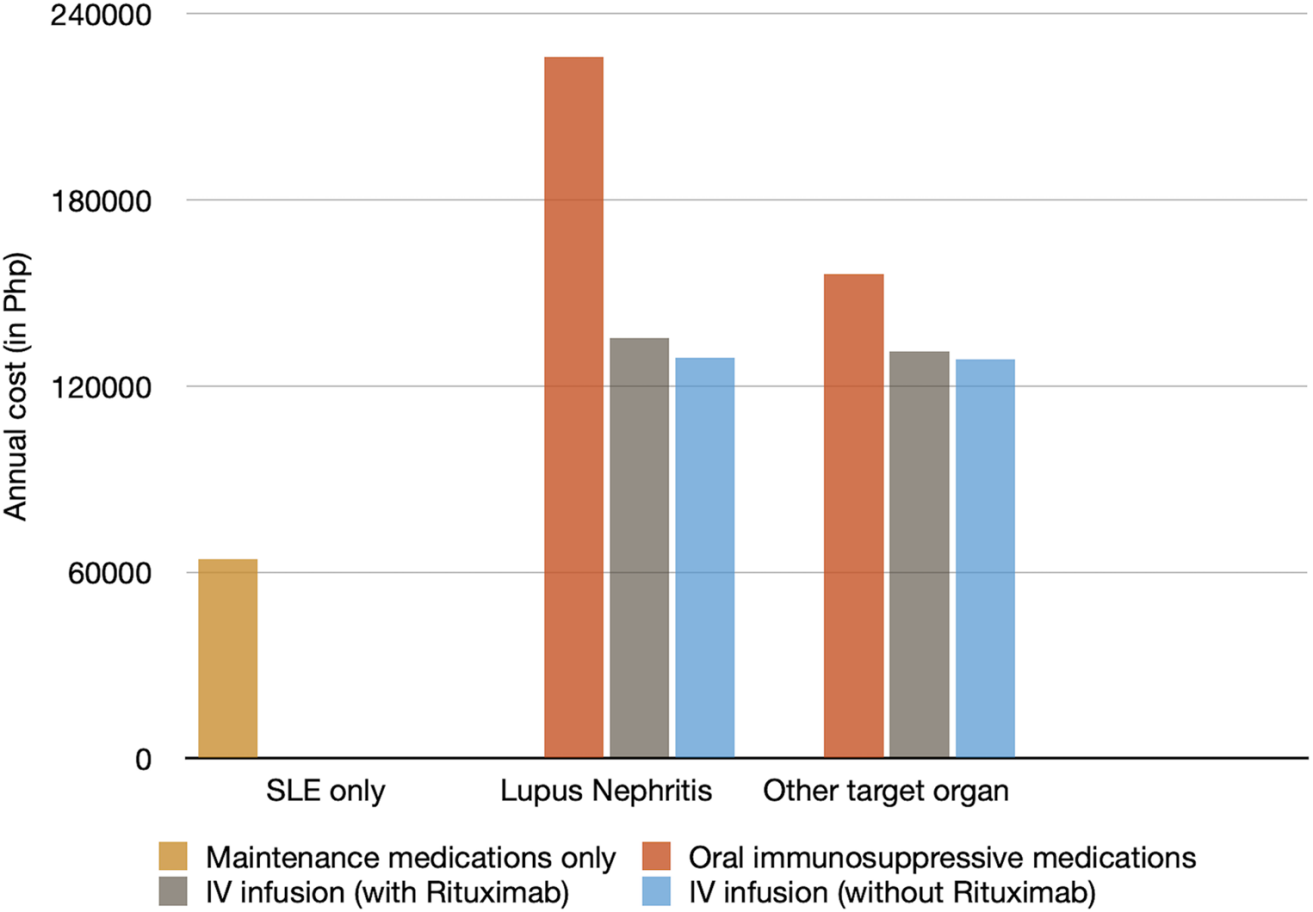
Mean annual direct cost categorized according to organ damage.

The annual direct medical costs of pediatric SLE patients are categorized according to the following: (1) pediatric SLE patients without target organ damage, (2) patients with lupus nephritis, and (3) those with target organ damage aside from nephritis. Patients with nephritis and other target organ damage have an increased cost of about 2–3.5× compared to those with no target organ damage, as shown in Figure 3.

**Figure 4:**
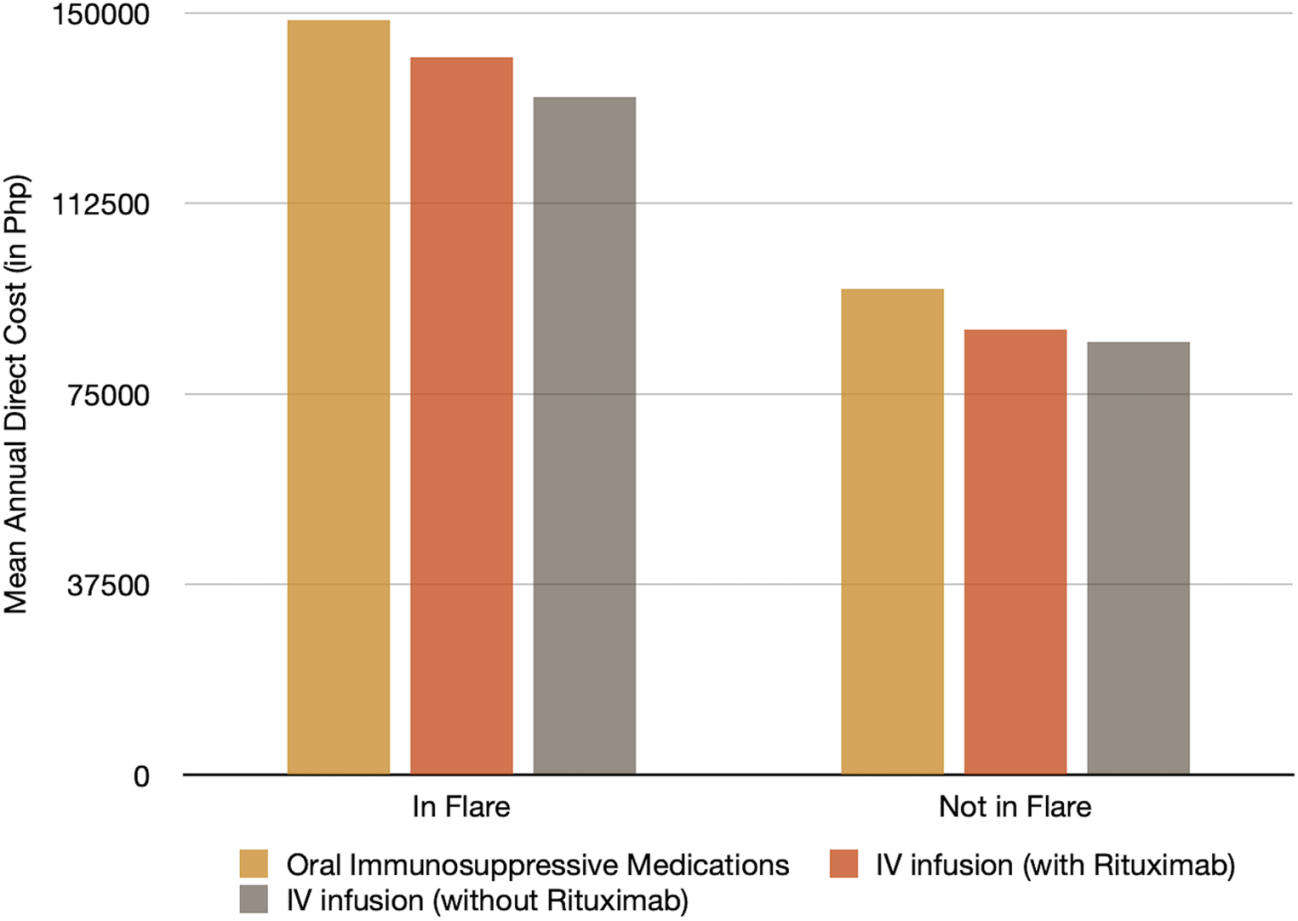
Mean annual direct cost for pediatric SLE patients in flare and not in flare. SLE, systemic lupus erythematosus.

In addition, costs between SLE patients in flare vs. those not in flare were compared; results showed a 1.6× increased cost for patients in flare compared to those not in flare as seen in Figure 4. The largest contributor to the increase in the cost for patients in flare was the need for additional immunosuppressive medications.

### Predictors of direct medical cost

A stepwise log linear multiple regression model was developed to identify predictors of cost in this study and is summarized in Table 5. The results were initially analyzed as a whole and overall, results showed that for every increase in the combined income of both parents, there is a corresponding increase in the direct cost for the doctor's fee per IV infusion (coefficient 3.329, *p*-value 0.01) and doctor's fee per clinic visit (coefficient 5.591, *p*-value 0.000).

**Table 5:** Stepwise log linear multiple regression analysis for cost predictors.

	Age		Sex		Disease duration		Organ involvement		Educational attainment		Combined income	
	Coefficient	*p*-value	Coefficient	*p*-value	Coefficient	*p*-value	Coefficient	*p*-value	Coefficient	*p*-value	Coefficient	*p*-value
SLE patients in flare												
Doctors’ fee for IV infusion	−0.127	0.9	−1.458	0.155	−1.469	0.151	−0.909	0.37	0.189	0.851	**2**.**109**	**0**.043*
Doctors’ fees for clinic visit	−0.82	0.418	−1.033	0.309	−0.433	0.668	0.561	0.579	0.85	0.402	**6**.**119**	**0**.000*
Oral immunosuppressive medications	−0.078	0.938	1.593	0.121	0.57	0.572	0.07	0.945	**−2**.**276**	**0**.030*	0.539	0.594
SLE patients not in flare												
Doctors’ fees for IV Infusion	−0.29	0.774	−0.213	0.833	−0.442	0.662	−0.551	0.586	0.2	0.843	**2**.**698**	**0**.011*
Doctors’ fees for clinic visit	−1.446	0.158	1.756	0.088	0.341	0.735	1.736	0.092	1.196	0.24	**2**.**319**	**0**.027*
Maintenance medications	**2**.**44**	**0**.020*	−0.167	0.868	−0.865	0.393	−1.561	0.128	0.464	0.645	0.855	0.399
	Age		Sex		Disease duration		Disease activity		Educational attainment		Combined income	
	Coefficient	*p*-value	Coefficient	*p*-value	Coefficient	*p*-value	Coefficient	*p*-value	Coefficient	*p*-value	Coefficient	*p*-value
SLE patients with lupus nephritis												
Doctors’ fees for IV infusion	−0.317	0.753	−1.131	0.263	−0.75	0.456	1.352	0.182	0.524	0.602	**3**.**294**	**0**.002*
Doctors’ fees for clinic visit	−1.073	0.288	0.999	0.322	−0.764	0.448	0.886	0.379	**2**.**181**	**0**.034*	**4**.**266**	**0**.000*
IV infusion/pulse therapy	0.688	0.495	**2**.**21**	**0**.031*	−1.539	0.13	1.072	0.289	1.482	0.144	−1.645	0.106
Maintenance medication	**2**.**452**	**0**.018*	−0.072	0.943	−1.372	0.176	0.84	0.405	0.851	0.398	0.458	0.649
SLE patients without lupus nephritis												
Doctors’ fees for Clinic visit	−1.397	0.186			1.911	0.078	1.36	0.197	0.707	0.492	**3**.**225**	**0**.007*

SLE, systemic lupus erythematosus.

Bold indicates *p*-value <0.05 is significant.

*p-value <0.05 is significant.

Results were further analyzed in subgroups, namely, those patients in flare and those who are not in flare, as well as patients with nephritis. Apart from the combined income of parents, results showed that age and educational attainment were cost drivers in this study. For patients in flare, lower educational attainment (coefficient −2.276, *p*-value 0.030) predicted a higher cost for oral immunosuppressive medications. For patients not in flare, the older the age of the patient, the higher the cost spent for maintenance medications (coefficient 2.440, *p*-value 0.020). For patients with lupus nephritis, the higher the educational attainment, the higher the cost spent for the doctor's fee for clinic visit (coefficient 2.181, *p*-value 0.034). Also, the older the age of the patient, the higher the cost spent for maintenance medications (coefficient 2.452, *p*-value 0.018). Female patients also predicted higher IV infusion costs (coefficient 2.210, *p*-value 0.031).

## Discussion

Cost of illness studies in SLE patients, particularly pediatric SLE patients, is very limited. In the author's knowledge, this is the first study on the direct cost of pediatric SLE patients in the Philippines.

The annual direct cost and the possible predictors of the increase in the cost were determined in this study. The mean annual direct medical cost of pediatric SLE patients in this study was estimated at 162,764.81 PHP (USD 3,047.23). This was compared to previous cost of illness studies and the results showed that it was similar to the local study done in the same center by Veñegas et al. (5) where the annual mean estimated cost was around USD 2,660, as well as the Korean study by Park et al. (6) where the cost was also estimated to be around USD 3,305. However, this result was significantly lower compared to US studies by Slawsiky et al. (7) and Brunner et al. (4) where they estimated the cost to be approximately USD 13,735–20,926 and USD 16,134, respectively. The wide range in healthcare cost in the previous studies may signify that comparison of direct healthcare cost with other countries is rather difficult due to differences in ethnicity, disease severity and manifestations, as well as differences in the cost per unit of the resources, healthcare system and utilization or due to differences in the methodology (7).

However, what may be consistent are not the actual sum of the costs but the trends in the results. This study showed that the largest component of the cost comes from medications which include IV infusions, maintenance, and oral immunosuppressive medications at 25.17%, 24.25%, and 18.10%, respectively. The result was similar to the previous studies where the largest component of healthcare expenditure came from medications as seen in the studies in the United States by Slawsky et al. (7) at 19%–30%, in South Korea by Park et al. (6) at 38.4%, and in Greece by Bertsias et al. (8) at 51.57%. The increase in the cost in Greece was attributed to the advent of novel immunosuppressive medications such as mycophenolate mofetil, azathioprine, and rituximab, the same medications being given to some of the patients in this study group.

Another comparable finding is that for patients with target organ damage, cost of illness was seen to exponentially increase, as seen in our study, where patients with nephritis and other target organ damage have an increased cost of about 2–3.5×. This was similar with the studies done by Cho et al. (9), Veñegas et al. (5 and Zhu et al. (10).

Among the 79 patients included in this study, 39 patients were in flare. Results showed that patients in flare had an increase cost of about 1.6× compared with those not in flare. This was similar to studies done in South Korea (6), Greece (8), and Sweden (11) where patients in flare accrued a higher cost (10). As seen in the study by Cho et al. (9), increase in the direct cost was attributed to the increase in the cost spent for oral immunosuppressive medications.

Based on the literature, SLE varies by ethnicity, and Asians tend to manifest more with hematologic, neurologic, and renal involvement (7). They are more predisposed to have a more active and severe disease manifestations compared with other ethnic groups. SLE cost is also expected to increase steadily over time due to preexisting disease manifestations plus other additional costs such as the adverse effects of treatment like prolonged steroid use (7). There are also differences in the cost per unit of medical resource per country, as well as variations in healthcare utilization. Patients in the United States are covered by insurance; hence, physicians are able to do the necessary tests since the costs are partly covered by their insurance company and are not entirely out of pocket like in the Philippines. As was encountered in the previous studies, results of this study were also highly skewed and variable. This may be due to the use of self-reported resource utilization rather than a claim-based analysis similar to other studies.

In the stepwise log linear regression analyzed to predict possible cost drivers, when analyzed as a whole, overall, the results showed that the higher the combined income of both parents, the higher the amount spent on the doctor's fee for clinic visits and IV infusions. Parents with a higher combined income may have more financial resource to allot for clinic visits and IV infusion therapy compared with those who earn less. Also, the setting of this study was in a teaching institution with both private and clinical divisions. In the private division, the professional fee of the doctor is significantly higher compared to the clinical division rate where patients are only charged minimally. However, in the clinical division, the cases of these patients are also shared to medical students. Hence, patients with more resources to allot for medical consults would usually choose the private division.

Apart from the combined income of the parents, when the results were further analyzed per subgroup, other cost drivers were the age of the patient and the parent's educational attainment.

For SLE patients and those with lupus nephritis, the older the age of the patient, the higher the cost spent for maintenance medications. This can be explained by the fact that pediatric medications are being computed by their weight.

In this study, patients with a lower educational attainment spent more on immunosuppressive medications. There is no direct explanation for this, but in a study by Barber et al. (12), education was used as a surrogate for disease activity. With this, there is a probability that poor compliance to medications or follow-up could have cost the flare, hence, eventually causing a higher cost for oral immunosuppressive medications. However, further investigation is needed.

For patients with lupus nephritis, a higher educational attainment predicted higher cost spent for clinic visits. Similarly, in the review done by Lau and Mak (13), healthcare cost was higher in those with higher education. The result was also congruent with the study done by Park et al. (6), where a higher educational level predicted a higher cost which they attributed to better follow-up and compliance with their doctors. This may also be due to a better understanding of the disease as well as a better grasp of the importance of close follow-up with their physicians.

Discrepancies in the mean annual cost were seen when compared with other studies, but overall, it is evident that pediatric SLE causes a significant economic burden on the patient and their families. In the Philippines, being a Third World country, where healthcare is not subsidized by the government, parents rely on their own financial resources to sustain the medical needs of their children. Nevertheless, we have seen how parents, despite difficulties financially, work hard to prioritize and secure medications for their children. Financial aid from government agencies has in the past helped this group of patients. However, in recent years, getting financial assistance from these agencies has been more difficult. Based on the present study, the government should make healthcare more accessible and sustainable for all. They should take an active role in alleviating the cost by creating subsidies for medications most especially biologic medications. Better control of their disease such as prevention of target organ damage and flares may substantially decrease the general cost spent for SLE.

The result of this study may give a better understanding of the economic burden of SLE patients, which may pave the way for the government to be aware of the high costs needed to maintain the disease. Awareness may subsequently lead to a more substantial budget allocation to achieve a better health outcome for this population and, thereafter, a stronger health system.

### Limitation points in this study

First, this study only included patients in a single center in Metro Manila. For future studies, a multicenter study that will include patients from government institutions or patients from other regions may be more favorable in order to get a better picture of pediatric SLE in the Philippines. A longer duration of the study may also aid in cost pattern analysis for pediatric SLE patients. A more comprehensive subcategory spent for healthcare may also help in the analysis. Second, the study used a self-reported questionnaire, which is subject to recall bias. However, in the Philippines, claims-based analysis may not be feasible as in other countries like the United States. All the same, a more accurate method of gathering the cost should be looked into.

## Conclusion

This is the first study that reported on the direct cost of pediatric SLE patients in the Philippines. For SLE patients with target organ damage like nephritis, cost was seen to increase up to 2–3.5×. For pediatric SLE patients who are in flare, cost was seen to increase up to 1.6×. Overall, the cost driver in this study was the parent's/caregiver's combined income. Further analysis into subcategories showed that the age and educational attainment also affected the cost spent for medications. Majority of the cost is attributed to the patient's medication.

## Data Availability

The original contributions presented in the study are included in the article, further inquiries can be directed to the corresponding author.
